# Enhancing Water Resistance and Mechanical Properties of Cemented Soil with Graphene Oxide

**DOI:** 10.3390/ma17071457

**Published:** 2024-03-22

**Authors:** Wei Lu, Xiaoqi Yan, Zhentao Bai, Dongbo Li, Chunsheng Lu

**Affiliations:** 1 School of Science, Xi’an University of Architecture and Technology, Xi’an 710055, China; luwei@xauat.edu.cn (W.L.); pengshif01@163.com (X.Y.); 2 National Key Laboratory of Green Building, Xi’an University of Architecture and Technology, Xi’an 710055, China; 3 School of Civil Engineering, Xi’an University of Architecture and Technology, Xi’an 710055, China; 4 School of Civil and Mechanical Engineering, Curtin University, Perth, WA 6845, Australia; c.lu@curtin.edu.au

**Keywords:** graphene oxide, cemented soil, water transport, unconfined compression strength, micromechanism

## Abstract

Although cemented soil as a subgrade fill material can meet certain performance requirements, it is susceptible to capillary erosion caused by groundwater. In order to eliminate the hazards caused by capillary water rise and to summarize the relevant laws of water transport properties, graphene oxide (GO) was used to improve cemented soil. This paper conducted capillary water absorption tests, unconfined compressive strength (UCS) tests, softening coefficient tests, and scanning electron microscope (SEM) tests on cemented soil using various contents of GO. The results showed that the capillary water absorption capacity and capillary water absorption rate exhibited a decreasing and then increasing trend with increasing GO content, while the UCS demonstrated an increasing and then decreasing trend. The improvement effect is most obvious when the content is 0.09%. At this content, the capillary absorption and capillary water absorption rate were reduced by 25.8% and 33.9%, respectively, and the UCS at 7d, 14d, and 28d was increased by 70.32%, 57.94%, and 61.97%, respectively. SEM testing results demonstrated that GO reduces the apparent void ratio of cemented soil by stimulating cement hydration and promoting ion exchange, thereby optimizing the microstructure and improving water resistance and mechanical properties. This research serves as a foundation for further investigating water migration and the appropriate treatment of GO-modified cemented soil subgrade.

## 1. Introduction

Cemented soil is an enhanced composite material created by uniformly blending water, cement, raw soil, and additional additives in specific proportions [1]. Due to its affordability and remarkable strength, cemented soil finds extensive application in engineering, particularly as a subgrade filling material [2]. Nonetheless, the extensive use of cement can lead to ecological pollution, thereby contributing to the heightened greenhouse effect and energy dissipation [3]. Post-cement curing, the soil exhibits a high alkalinity, significantly impacting both groundwater quality and plant development. Additionally, in low-lying regions, the cemented soil subgrade is vulnerable to erosion from groundwater. This erosion primarily occurs due to water migration facilitated by the capillary effect, resulting in a reduction in soil strength. Accordingly, there is a need to explore environmentally friendly additives that demonstrate substantial resistance to water corrosion.

In recent years, there has been an increasing interest in employing nanomaterials as reinforcing agents in composite materials, owing to their exceptional properties. These materials have shown great potential for use in engineering construction and are expected to have a significant impact on the field [4]. Previous studies have demonstrated that the incorporation of an optimal quantity of nanomaterials into cemented soil can greatly improve and enhance its microstructure and mechanical properties. It is shown that the optimal quantity of SiO_2_ can significantly enhance both the hydraulic conductivity and compressive strength of cemented soil [5]. The highly reactive nano-SiO_2_ can efficiently catalyze the formation of calcium silicate hydrate gels (C–S–H) and facilitate their uniform dispersion [6]. Using nano-magnesia–cement, the cured seashore soft soil had a significant increase in shear strength and shear modulus [7]. The mechanical properties of cemented soils amended with nano-CaCO_3_ were explored in marine environments. These findings revealed that the incorporation of nano-CaCO_3_ stimulated the formation of hydration products, leading to improved compressive strength and corrosion resistance [8]. Nanomaterials such as nano-calcium carbonate (NC), nano-graphene oxide (NG), nano-silicon dioxide (NS), and nano-titanium dioxide (NT) can be used to modify cement slurry, and different nanomaterials promote the formation of different hydration products, thereby improving the early strength of cement [9].

When considering nanomaterials, graphene oxide (GO) emerges as a novel type possessing a multitude of highly active functional groups on its surface. This material exhibits outstanding mechanical properties and hydrophilicity and finds extensive application in the fields of metal reinforcement and cement-based materials [10]. Nevertheless, to the best of our knowledge, there are few studies on using GO to enhance cementitious soil. For example, the feasibility of GO for improving the performance of cemented soil was verified through compaction tests, unconfined compressive strength (UCS) tests, and direct shear tests [11]. The effect of GO on the compactness and mechanical properties of the cemented soil was investigated through experiments, and the results showed that GO had a significant enhancement effect on the mechanical properties and microstructure of the cemented soil [12].

Relevant studies have shown that the production mode of GO is constantly improving, which shows that the cost of GO can be further reduced [13,14], and the wide use of graphene concrete can significantly reduce the carbon footprint of the cement industry. Cement production accounts for more than 70% of carbon emissions from concrete. According to current estimates, graphene concrete can reduce emissions by 18–20% and cement content by 20–50%. In addition, studies have shown that GO can be used to treat harmful substances, which is beneficial to the ecological environment and human health [15,16,17].

The above studies have enriched the progress of nanomaterials in improving soil engineering properties, but there are few studies on the weakening characteristics of sewage erosion and the migration rule of capillary water in cemented soil road bases. Therefore, this paper explored the water transport and compressive properties of modified cemented soil, leveraging the exceptional performance of GO. The impact of GO on the microstructure of cemented soil was investigated, and its enhancement mechanism was examined. The analysis of water migration within the GO-improved cemented soil subgrade provides researchers with valuable insights on how to effectively treat and maintain this material for optimal performance.

## 2. Test Materials and Methods

### 2.1. Materials

**Figure 1:**
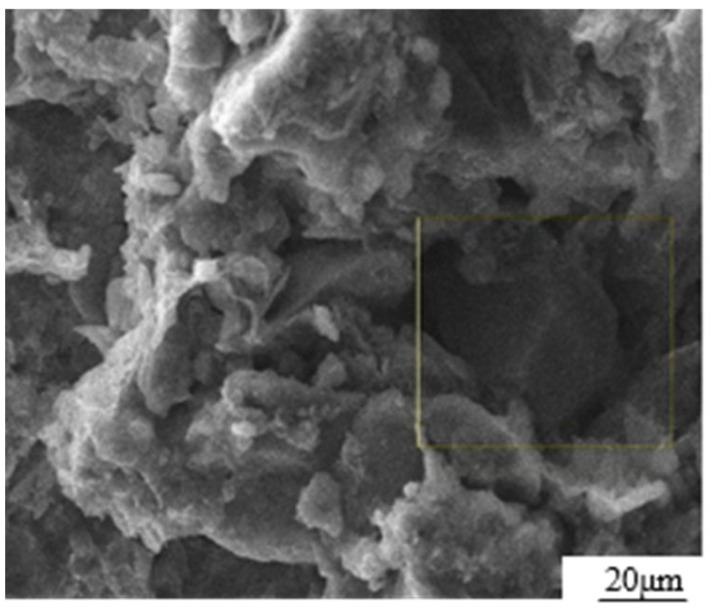
SEM photograph of loess microstructure.

The test materials consist of loess, GO dispersion, and ordinary silicate cement. The loess material was collected from Baqiao District in Xi’an City, Shaanxi Province. The main chemical composition and physical properties are listed in Table 1 [18]. The SEM photograph, as shown in Figure 1, clearly illustrates that the particles were coated with colloidal substances, while the colloidal substances themselves were filled with particles. This observation aligned with the typical microstructure features of loess during the middle and late stages of the Quaternary period. The particle composition of loess is composed of powder particles (5–50 μm, 68%), followed by clay particles (<5 μm, 26%) and sand particles (>50 μm, 6%).

The cement utilized here was P.O 42.5 ordinary silicate cement, produced by Tong Chuan Dongguan Cement Co., Ltd. (Dongguan, China). The principal constituents of the cement are presented in Table 2. The GO dispersion employed in this study was of the SE3522 type, manufactured by Changzhou Sixth Element Material Technology Co. (Changzhou, China). The primary constituents of the GO dispersion are listed in Table 3.

### 2.2. Specimen Preparation

Table 4 displays the water content, cement content, and GO content of the specimens. The water–solid ratio represents the mass ratio of water to solid material; the cement mixing ratio signifies the mass ratio of cement to loess; and the GO admixture corresponds to the mass ratio of GO with cement.

**Figure 2:**
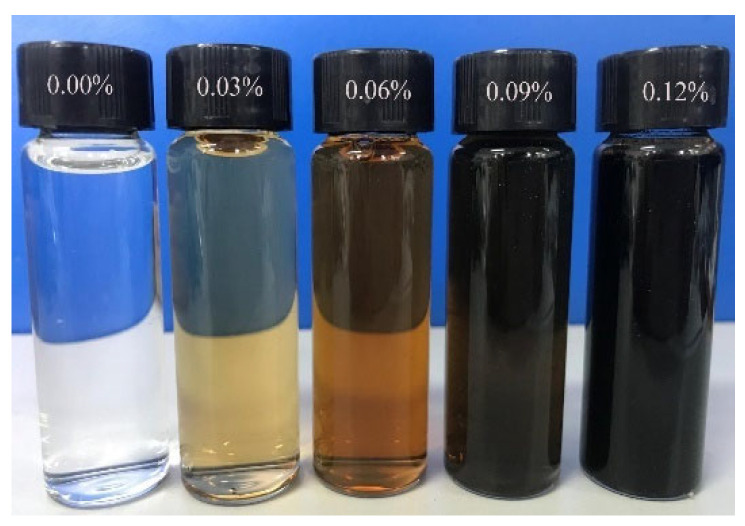
GO aqueous solutions after ultrasonic dispersion.

**Figure 3:**
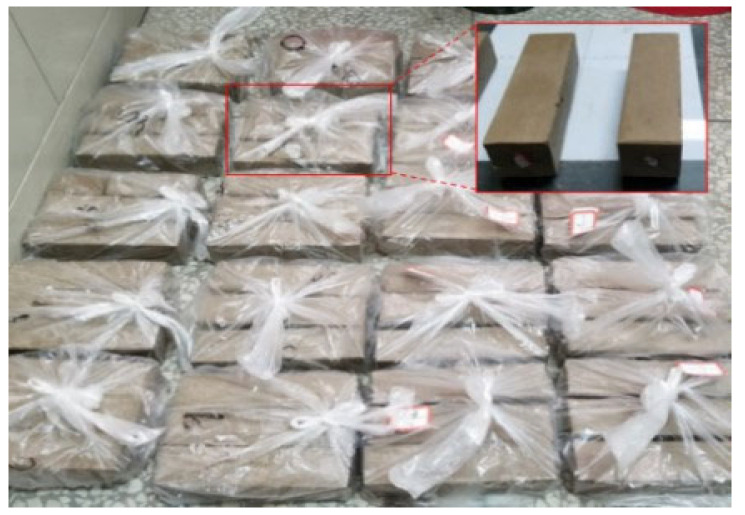
Diagram for preparation of specimens.

The collected natural soil was air-dried outdoors to reduce moisture and then crushed with a rubber hammer. The particle size was sieved through a 2 mm sieve to eliminate oversized particles in the experiment. The GO, diluted to the specified concentration, was subjected to 1 h of ultrasonic dispersion to acquire a uniformly dispersed GO solution (Figure 2). The mixture was homogeneously mixed in compliance with the concrete admixture standard [19]. The cement was blended with water, added to the sonicated GO solution, and stirred for 2 min. It was then combined with the remaining loess and continuously stirred for another 2 min. Cuboid test blocks measuring 40 × 40 × 160 mm^3^ were fabricated, compacted in two layers, and subsequently covered with plastic film for 48 h before demolding (Figure 3). Under controlled conditions of 20 ± 2 °C and 60–70% relative humidity, the specimens were cured to reach the desired test age.

### 2.3. Test Methods

#### 2.3.1. Capillary Water Absorption Test

**Figure 4:**
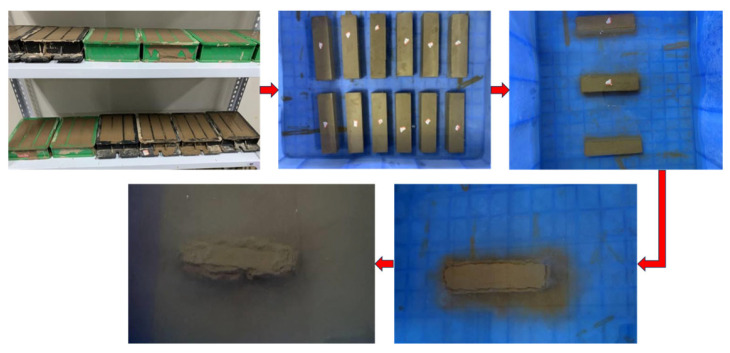
Schematic diagram of capillary water absorption test.

Based on the UCS test method in JTG E51-2009 [20] “Test Methods of Materials Stabilized with Inorganic Binders for Highway Engineering”, specimens were dried and coated with epoxy resin on 5 sides to prevent water penetration, leaving only 1 bottom side for the water absorption test (Figure 4). Prior to testing, the initial weight of the specimen (*M*_0_) was recorded. During the test, the specimens were removed at specific intervals (5 min between 0 and 60 min, 15 min between 60 and 360 min, and 30 min between 360 and 540 min) to measure their weight. Excess water from the surface of the specimen was wiped away before weighing it until its mass no longer changed, which is referred to as the saturation mass (*M*_1_). The water absorption can then be determined as follows:

(1) $$M=M_{1}-M_{0}$$

where *M* is the water absorption, *M*_1_ is the mass of the specimen after saturation with water, and *M*_0_ is the initial mass of the specimen.

#### 2.3.2. UCS Test

The UCS test was carried out according to [21]. The UCS of a specimen was tested at 7d, 14d, and 28d using an INSTRON 5848 material testing machine (Instrong (Shanghai) Test Equipment Trading Co., Ltd., Shanghai, China) and a 40 × 40 mm^2^ special indenter, loaded uniformly at a rate of 1800 ± 200 N/s. The UCS is calculated as follows:

(2) $$\sigma_{c}=\frac{F_{e}}{A}$$

where *σ*_c_ is the UCS; *F*_e_ is the peak load at the damage of the specimen; and *A* is the compressed area, 40 × 40 mm^2^.

#### 2.3.3. Softening Coefficient Test

Following the “Standard for test method of mechanical properties on ordinary concrete” GB/T50081-2019 [22], the two sets of specimens were formed simultaneously using the same ratio. In the first set, the compressive strength was tested directly after 28 days of curing and recorded as *I*_0_. In the second set, specimens were immersed in water with the water surface positioned 100 mm above the specimens following a 28-day curing period. The UCS was then tested after 24 h and recorded as *I*_1_. The softening coefficient can be determined as follows:

(3) $$K=\frac{I_{1}}{I_{0}}$$

where *K* is the softening coefficient, characterizing the water resistance of the material. The greater the softening coefficient, the better the water resistance.

#### 2.3.4. Microstructure Tests

The SEM test was performed using a scanning electron microscope (model: HITACHI SU8010, Snetar Precision Instruments (Shanghai) Co., Ltd., Shanghai, China) for microstructure testing at a magnification level of 1000×. Specimens were extracted from the central region of a 28-day UCS test specimen. They were obtained as flakes with an approximate area of 1 cm^2^ and a thickness measuring 2 mm. Prior to the test, the specimens were subjected to freeze-vacuum drying to achieve complete sublimation of any water contained within the pores. To render the sample surface conductive and capable of reflecting secondary imaging, the flake sample under preparation must be coated with a layer of gold. Throughout the SEM test, the instrument voltage was adjusted to a voltage of 20 kV. Following a precise adjustment of the focal length, the overall morphology of the sample was observed.

## 3. Results and Discussion

### 3.1. Characterization of GO

**Figure 5:**
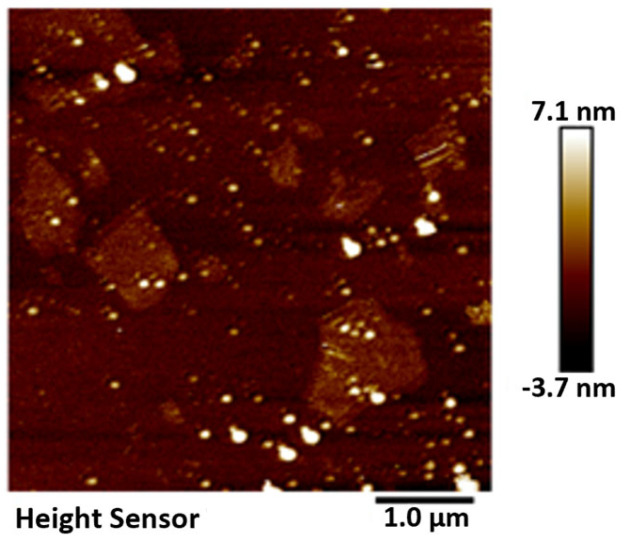
AFM characterization of GO.

**Figure 6:**
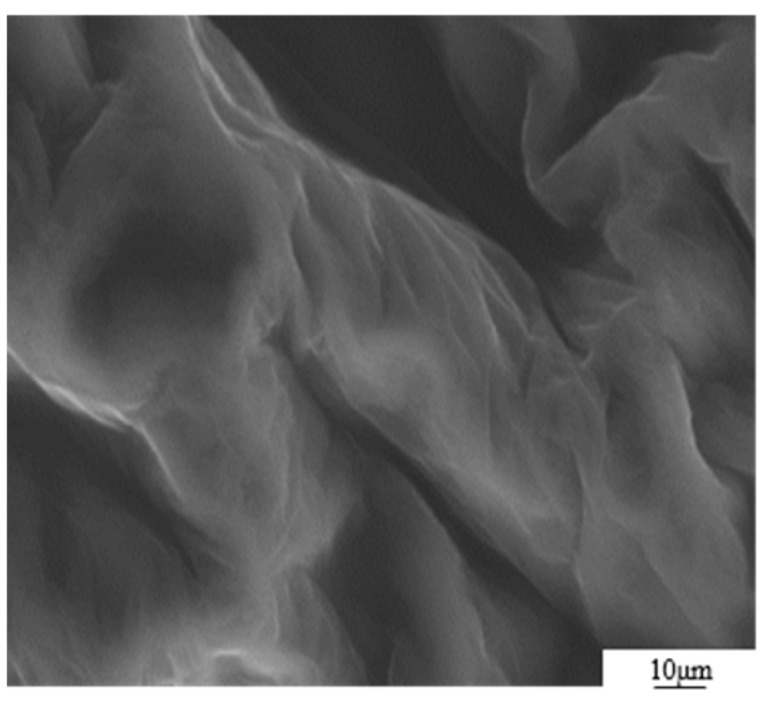
SEM image of GO sheet.

**Figure 7:**
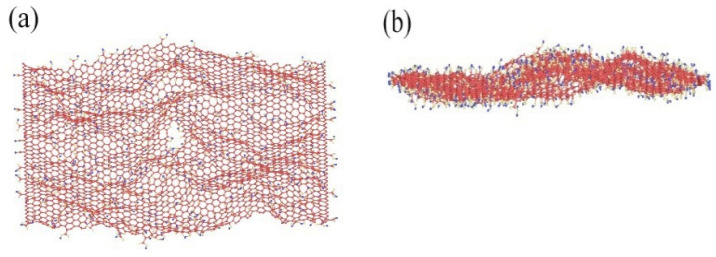
MD images of rough and wrinkled surfaces under (**a**) vertical view and (**b**) lateral view.

The GO was analyzed using atomic force microscopy (AFM). As depicted in Figure 5, the GO flakes displayed an average thickness of approximately 1 nm, suggesting that the GO flakes had achieved a thickness equivalent to that of a single atomic layer. The analysis of the particle size distribution of GO was conducted through the utilization of a laser particle sizer, as presented in Table 5. The obtained results exhibited a normal distribution, with a median particle size (D50) measuring less than 5.0 μm. Figure 6 presents an electron microscope image of GO, illustrating its silk-like folded appearance, which can be attributed to the substantial specific surface area of GO [23]. On the other hand, the formation of new sp_3_ hybridization states during the preparation of GO, coupled with the introduction of numerous intrinsic and topological defects, results in the occurrence of irregular folds in the surface layer of GO (see Figure 7) [24]. The rough and wrinkled surface of the GO additionally contributes to a strong mechanical interlocking between the GO and the substrate, thereby improving the interfacial mechanical properties [25]. In comparison to the 3.38 nm layer spacing of graphite, the layer spacing of GO expands to 8.04 nm as a result of the introduced functional groups. These functional groups increase the spacing between layers and weaken the hydrogen bonding forces. Consequently, this augmented layer spacing promotes the homogeneous dispersion of GO in both water and within the matrix [26].

### 3.2. Effect of GO Content on the Capillary Water Absorption of Cemented Soil

**Figure 8:**
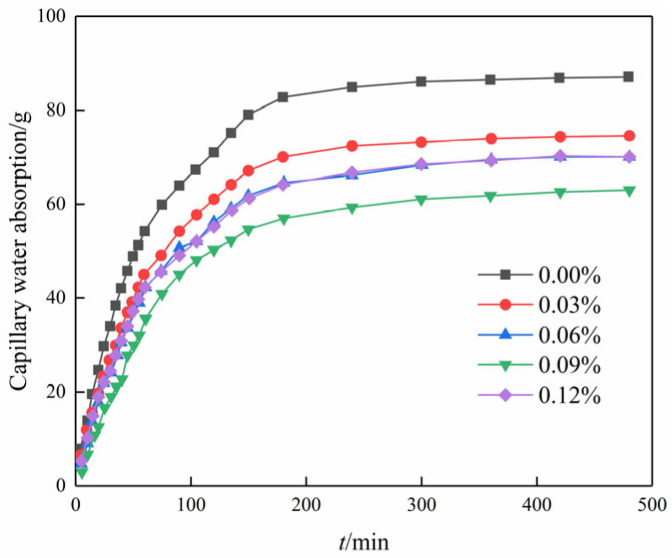
Capillary water absorption curve of modified cemented soil with time.

Figure 8 illustrates the variation in capillary water absorption over time for different contents of GO. It is seen that all curves are in the shape of an overall parabola. The parabolic curves, representing various GO contents, exhibit three distinct stages: (1) During the initial stage of the experiment, the data exhibit an almost linear trend, indicating a consistent increase in the amount of water absorbed by the soil. Additionally, the slope of the line representing this trend is large, which indicates that the rate of absorption is high. (2) Between 80 and 220 min into the experiment, the slope of the line representing the trend in the data no longer changed linearly. This means that the rate of water absorption was no longer increasing at a steady rate but rather at a changing rate. However, despite the altered slope, capillary water absorption continued to progressively increase over time. (3) After 220 min into the experiment, it is observed that the capillary water absorption maintains a near-constant level. This observation suggests that the specimen has reached saturation, indicating that it is no longer capable of further water absorption.

Moreover, the capillary water absorption showed a trend of decreasing and then increasing with the increase in GO content in the same period, and the water absorption was the smallest when the content was 0.09%. The saturation water absorption decreased by 28.3% in comparison to the blank specimen. The inclusion of GO influenced the compact aggregation of crystals, leading to a tendency for further densification and interweaving. Therefore, adding GO to the soil–cement matrix resulted in a decrease in the pore volume in the soil sample and the formation of a compact structure [27]. Subsequently, the water absorption increased rather than decreased with an increase in the content of GO. This is attributed to inadequate dispersion within the matrix at excessive levels of GO doping, thus compromising its reinforcing effect on the matrix material [28].

**Figure 9:**
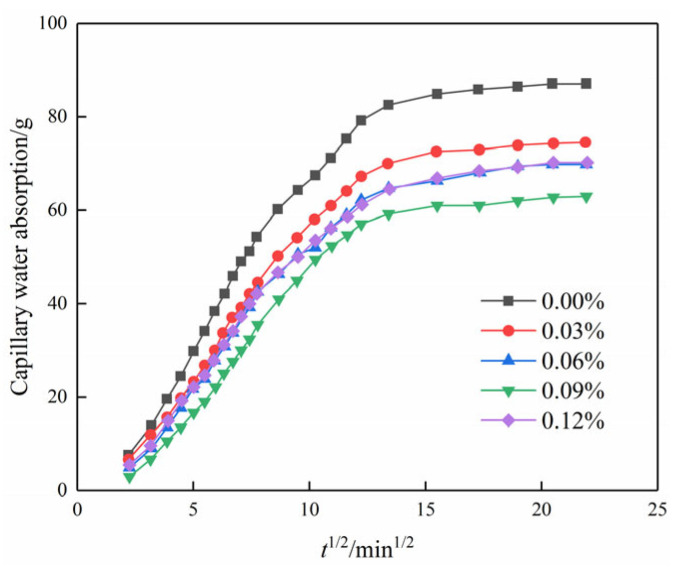
Capillary water absorption of modified cemented soil as a function of time square root.

Figure 9 illustrates the relationship between capillary water absorption and the square root of time. It is evident that the water absorption in the initial stage exhibits a linear correlation with the square root of time. This suggests that not only do chemical modifiers such as cement follow the “square root of time” law for capillary water absorption mass increment in porous materials but that nanomaterials such as GO follow a similar law for the modification of plain soils [29].

**Figure 10:**
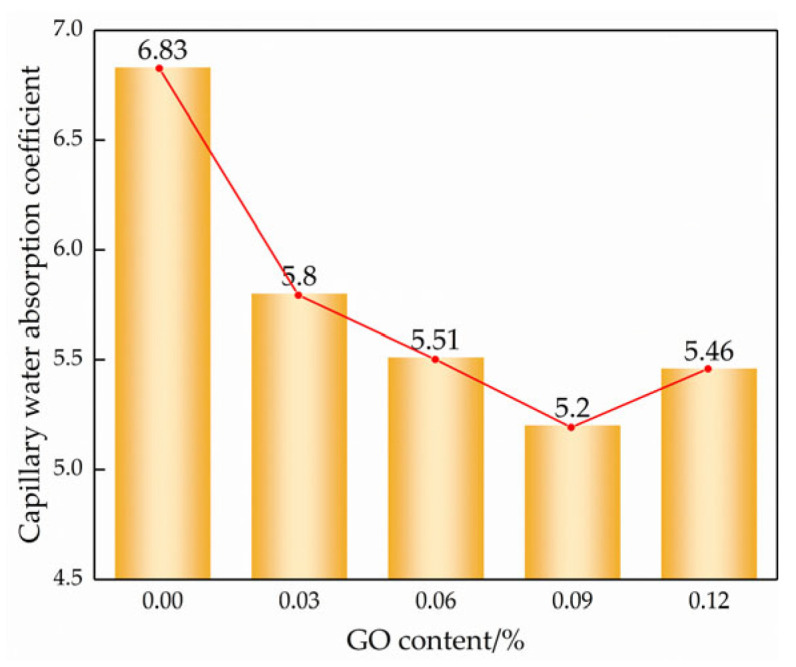
Capillary absorption coefficient of different GO contents.

**Figure 11:**
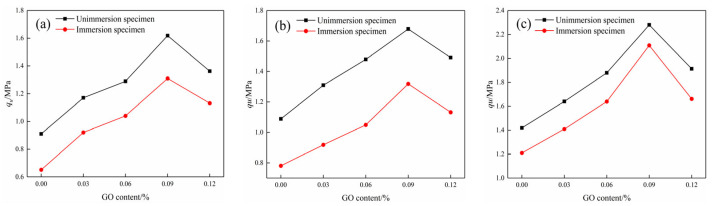
Compressive strength of specimens before and after water immersion at different curing ages: (**a**) 7d compressive strength, (**b**) 14d compressive strength, and (**c**) 28d compressive strength.

According to the “square root of time” law for porous materials [29], the slope can be divided into a linear stage and a stable stage in Figure 10, where the slope of the linear stage is the capillary absorption coefficient of the modified cemented soil. The slope of the line for each GO content is the capillary water absorption coefficient according to the linear regression equation, as shown in Figure 11.

As observed in Figure 9 and Figure 10, the incorporation of GO noticeably improved the water transport characteristics of the cemented soil, resulting in a reduction in both capillary water uptake and the capillary water absorption coefficient. Compared to the control specimens, the capillary absorption coefficients were reduced by 14.6%, 19.5%, 25.8%, and 21.3% at GO contents of 0.03%, 0.06%, 0.09%, and 0.12%, respectively. This demonstrated that GO exerts a notable inhibitory effect on capillary water absorption in hydric soils. The inhibitory effect of GO content showed a strong threshold characteristic: it increased with increasing GO content up to 0.09% but decreased thereafter.

### 3.3. Effect of GO Content on the Capillary Water Absorption Rate of Cemented Soil

Table 6 lists the capillary water absorption rates of the modified cemented soils at different time intervals. It clearly demonstrates that the inclusion of GO leads to a significant reduction in the capillary water absorption rates of the cemented soils across different stages. The inclusion of GO at content levels of 0.03%, 0.06%, 0.09%, and 0.12% resulted in water absorption rate reductions of 16.5%, 21.3%, 33.9%, and 22.3%, respectively, compared to the blank specimens. It is seen that GO content had a significant effect on the water absorption rate and also showed the threshold characteristic. When the content was 0.09%, GO had the greatest effect on the capillary water absorption rate and the most obvious reduction.

### 3.4. UCS Test Results

The UCS tests were conducted on the modified cemented soil specimens to assess their mechanical properties before and after water immersion at different ages. As presented in Figure 11, the UCS of the test piece increased first and then decreased with the increase in GO content at different curing ages. When the content of GO was 0.09%, the compressive property was the highest. After water absorption, the UCS of specimens at 7d, 14d, and 28d increased by 70.32%, 57.94%, and 61.97%, respectively, when compared to the blank specimens. Furthermore, the compressive strength of specimens decreased after water immersion compared to the non-immersion specimens at the same age, suggesting that water erosion significantly impairs the mechanical properties of cemented soils [30].

**Figure 12:**
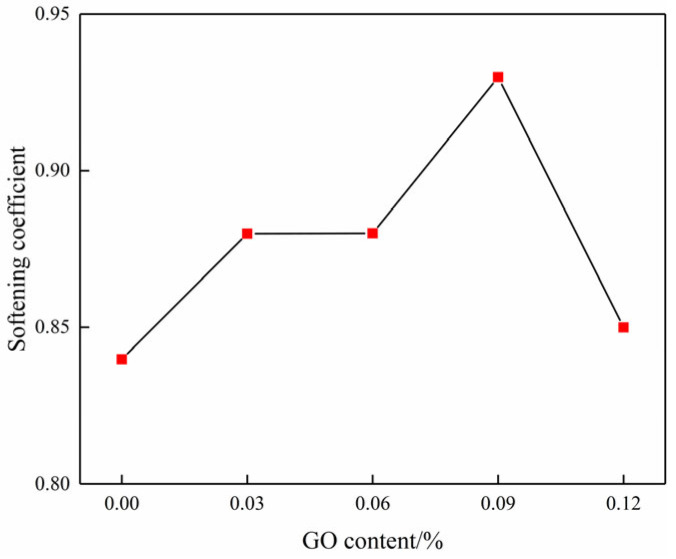
Curve of the softening coefficient changing with GO content.

According to Equation (3), the variation in the softening coefficient as a function of GO content can be further obtained, as shown in Figure 12. It clearly illustrates that the softening coefficient exhibited a gradual increase as the GO content was raised from 0% to 0.09%, resulting in increases of 4.76%, 4.76%, and 10.16% when compared to the blank specimens. Nevertheless, the softening coefficient shows a downward trend beyond a GO content of 0.09%, suggesting that a suitable amount of GO can greatly enhance the water resistance of cemented soil [31].

## 4. Mechanistic Analysis of GO-Modified Cemented Soils

### 4.1. Microstructural Analysis

**Figure 13:**
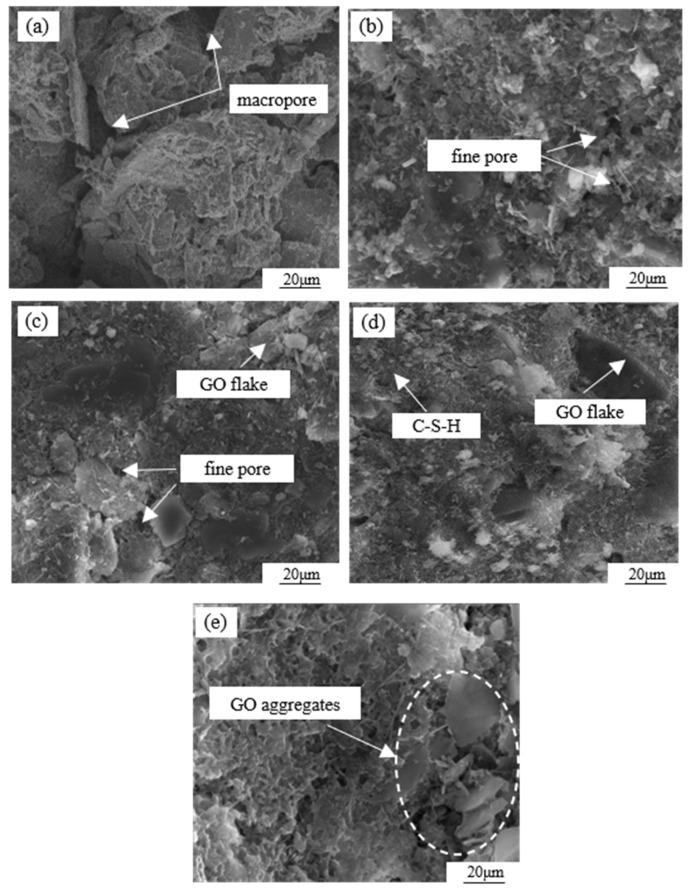
SEM scanning images of cemented soil materials with different GO contents: (**a**) 0.00% GO, (**b**) 0.03% GO, (**c**) 0.06% GO, (**d**) 0.09% GO, and (**e**) 0.12% GO.

To further analyze the mechanism of GO modification on the cemented soil, SEM tests were conducted on specimens with different GO contents, as shown in Figure 13. In the absence of GO admixture (Figure 13a), the soil particle surfaces were coated with hydrates formed during cement hydration. However, there was a lower amount of cement filling the gaps between the particles, resulting in an overall loose skeleton characterized by a structure of large particles and large pores. From Figure 13b, it is evident that at a GO content of 0.03%, there was an increase in the quantity of spherical and needle-like small particles present in the sample. These particles were distributed on the larger particles, effectively filling the pores and establishing surface-to-surface contact. However, due to the limited quantity, the pores cannot be completely filled, showing a large particle–small particle–micropore–soil particle structure. When the GO content further increased to 0.06% (Figure 13c), the number of small particles increased significantly and then aggregated to form aggregates that filled pores and compacted the soil. This forms a structure composed of large particles–aggregate particles and soil particles. As shown in Figure 13d, when the GO dose is 0.09%, the number of small particles continues to increase, pores are effectively filled, and particles are tightly combined to form a dense structure. Figure 13e demonstrates that a GO content of 0.12% resulted in the reemergence of larger pores within the microstructure, accompanied by a decrease in inter-particle cements, resulting in an overall porous structure.

Based on the aforementioned analysis, there exists a critical threshold for the microstructural density of GO-modified specimens in relation to the amount of GO admixture. Excessive or insufficient admixture is found to adversely affect the structural compactness. When mixed solely with cement, the hydration products are limited to coating the surface of larger particles, resulting in a weak cementing effect and the presence of a significant pore structure [32]. The hydration process of Portland cement involves a multi-phase and intricate chemical reaction. This reaction facilitates the transformation of dispersed cement powder particles into a binding cement slurry, generating a variety of hydration products and effectively amalgamating particles of varying sizes [33]. Particularly during the initial stages of hydration, the prevalent hydration products include tetracalcium aluminoferrite (4CaO·Al_2_O_3_·Fe_2_O_3_) and tricalcium aluminate (3CaO·Al_2_O_3_). However, during this period, the resulting hydrate exhibits negligible effects on the enhancement of the cemented soil and displays low density [34].

**Figure 14:**
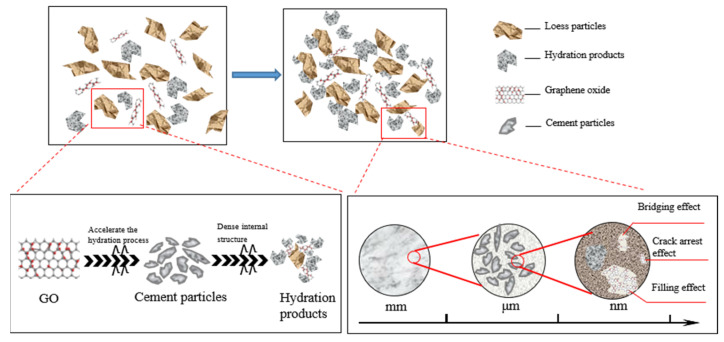
Schematic diagram of the mechanism of GO-reinforced soil–cement material.

Figure 14 presents a schematic diagram illustrating the mechanism analysis of GO-enhanced cemented soil. It is shown that the increase in GO dose has two different effects. First, GO can effectively fill micropores and microcracks in the soil, so as to improve the compactness of the structure. In addition, GO also acts as a bridging agent to further enhance structural integrity. Finally, the surface of GO is rich in active functional groups, including hydroxyl, carboxyl, and epoxy groups. These functional groups have the ability to stimulate the hydration reaction of cement, facilitating the interweaving of calcium silicate hydrate (C–S–H) and other products, resulting in the formation of a network-like structure. Consequently, this process enhanced the integrity and compactness of the structure [3].

The primary constituent of cement hydration products is the calcium silicate hydrate gel, which comprises approximately 50% to 60% of the total volume of hardened cement [35]. The C–S–H gel is essential for providing gelling strength and influencing significant engineering properties, including strength, shrinkage, and permeability resistance. It can exist in various forms, such as mesh-like, colloidal, or flocculent materials. The structure and properties of C–S–H gels play a crucial role in determining the fundamental engineering properties of gelled materials [36].

A 0.09% GO content resulted in increased hydrophilic and dispersive qualities within the soil–cement matrix after 28 days of curing, attributed to the presence of C, H, and O functional groups [31]. The cement with GO, when cured for 28 days, adapted to micropores and microcracks in the soil–cement matrix. The high surface area, two-dimensional properties, and pore-filling characteristics of GO contribute to the nucleation of hydration products and enhance the cement hydration process [37]. In this way, GO maintained a uniform structure and controlled the hydration process at the nanoscale [38]. The uniform distribution of GO prevented the agglomeration or clustering of cement in the soil–cement matrix. Consequently, the soil sample exhibited a positive effect by increasing the density and the number of hydration products, resulting in a polyhedral structure.

When the content of GO exceeds the critical value, the remaining GO flakes will adsorb a significant amount of free water. Consequently, this would reduce the availability of water for cement hydration, leading to inadequate hydration. On the other hand, the extensive specific surface area of GO resulted in a pronounced agglomeration effect. This hindered the uniform dispersion of GO within the cemented soil, thereby limiting its interaction with soil particles and reducing its effectiveness. Consequently, this led to a decrease in the formation of C–S–H [11].

### 4.2. Ion Exchange Effect

**Figure 15:**
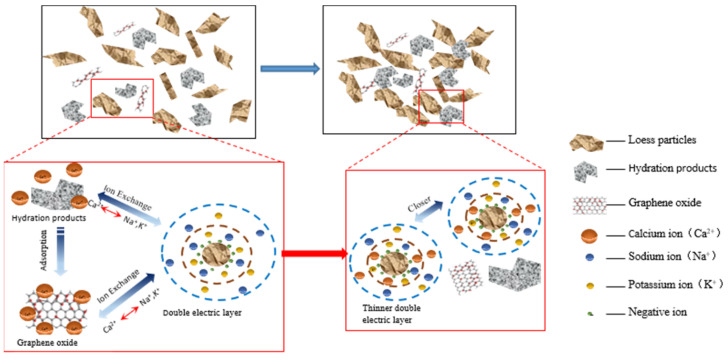
Schematic diagram of the GO-enhanced ion exchange effect of cemented soil.

In addition, the soil particle colloids are negatively charged and have substantial amounts of adsorbed K^+^ and Na^+^ on their surfaces. The addition of cement resulted in the production of Ca^2+^ during the hydration process. The Ca^2+^ ions undergo cation exchange with the K^+^ and Na^+^ ions present on the surface of soil particles. This exchange leads to a decrease in the thickness of the double electric layer and an increase in the bonding force between the soil particles. As a result, the microstructure of the soil experienced some improvement. Furthermore, the addition of GO greatly stimulates the cement hydration reaction, resulting in the generation of a substantial quantity of hydration products and Ca^2+^. This further enhances the exchange of Ca^2+^ with K^+^ and Na^+^ on the surface of the soil particles, resulting in a more pronounced thinning of the soil particles’ double electric layer and a significant increase in the bonding force between them (see Figure 15). This results in a stronger and denser overall structure, leading to a significant improvement in water resistance and mechanical properties [39].

### 4.3. Analysis of the Correlation between Microstructure and Macroscopic Properties

Although SEM images can demonstrate the differences between cement-treated soil modified with different amounts of GO from the perspective of micromorphology, it is difficult to quantitatively characterize microstructural indicators, such as apparent pore ratio, as well as the relationship between microstructure and macroscopic properties.

Particle (pore) and fracture image recognition and analysis systems for automatic identification, quantification, and statistical analysis of particles, pores, and fractures are based on optical microscopy and electron microscopy images [40]. The fundamental principle entails automating the removal of stray points through binarization, segmenting, and identifying particles and pores based on their respective grayscale values and conducting quantitative statistical analysis of microstructural parameters, including the apparent pore ratio and fractional dimensional values of porosity. Quantitative characterization of microstructures can be achieved through the above steps and test methods [41].

The apparent pore ratio is an essential indicator of soil density degree. This approach indirectly addresses the diverse characteristics of the pore ratio in three-dimensional space by utilizing two-dimensional parameters. The apparent pore ratio can be computationally characterized by calculating the ratio of pore area to soil particle area in the binarized image [42]. The apparent pore ratio is determined as follows:

(4) $$N=\frac{A_{p}}{A_{s}}$$

where *N* is the apparent pore ratio; *A*_p_ is the area of pores in the SEM images (μm^2^); and *A*_s_ is the area of soil particles in the SEM images (μm^2^).

**Figure 16:**
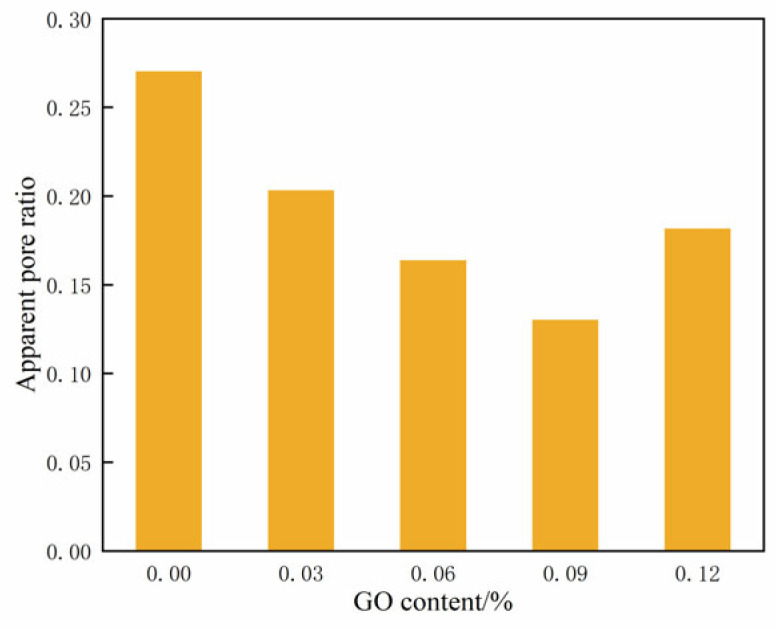
Apparent pore ratio of soil-cement under different GO contents.

Figure 16 displays the changes in the apparent pore ratio across varying contents of GO. It illustrates that the apparent pore ratio of the specimens initially decreased and then increased as the GO content increased. At a content of 0.09% GO, the apparent pore ratio reached its minimum value, indicating the highest level of compaction in the cemented soil.

**Figure 17:**
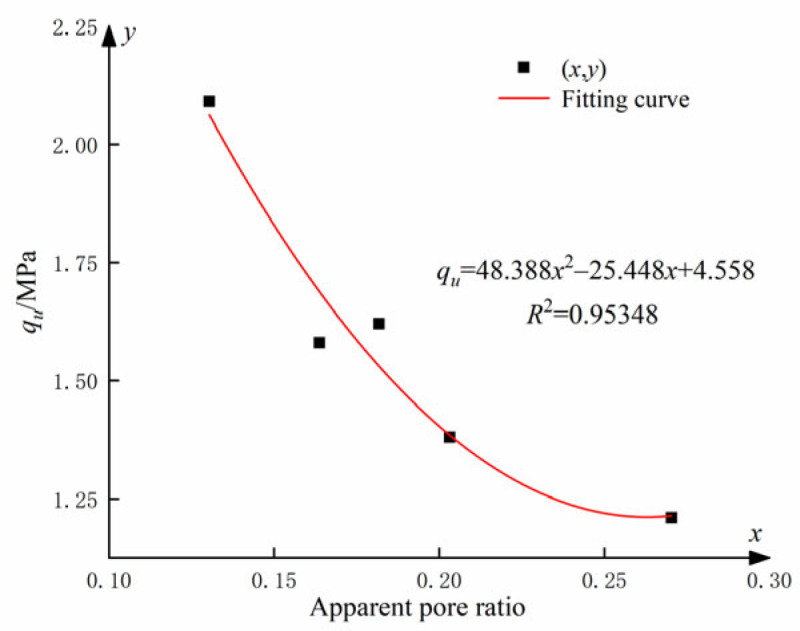
Apparent void ratio of soil-cement under different GO contents.

This analysis reveals a clear correlation between the UCS and the apparent pore ratio. Figure 17 illustrates the relationship between UCS and apparent pore ratio for cemented soils incorporating different levels of GO admixtures.

The relationship between the UCS and the apparent pore ratio *q_u_* (see Figure 17) can be fitted as follows:

(5) $$q_{u}=48.388x^{2}-25.448x+4.558$$

where *x* is the apparent pore ratio.

Equation (5) demonstrates a quadratic polynomial relationship between the UCS and the apparent pore ratio *q_u_*. The synergistic effect of an optimum amount of GO and cement stimulated the hydration process, resulting in the production of more abundant hydration products and the formation of numerous regular structures in the soil. Additionally, the hydration products have the ability to fill the voids between soil particles, whereas GO can penetrate even the smaller cracks. The synergistic combination of these two processes significantly enhanced the structural strength and compactness of the soil.

## 5. Conclusions

This paper has investigated the water transport properties, compressive properties, and modification mechanism of GO-modified cemented soils and verified that GO can be used to improve soil-cement and alleviate the harm caused by capillary water rise [12,43]. The main conclusions can be drawn as follows:

(1) The addition of GO significantly decreases the capillary water absorption, capillary water absorption coefficient, and initial capillary water absorption rate of the cemented soil. Furthermore, the modification effect of GO on the soil exhibits evident threshold characteristics. The optimal modification effect was achieved at a GO content of about 0.09%, resulting in a 25.8% reduction in the capillary water absorption coefficient and a 33.9% reduction in the initial capillary water absorption rate compared to the blank specimens.
(2) The addition of GO significantly enhanced the UCS of the cemented soil after water immersion, with the optimal effect observed at a content of about 0.09%. The UCS at 7d, 14d, and 28d increased by 70.32%, 57.94%, and 61.97%, respectively.
(3) The SEM test results revealed that the modification mechanism of GO on cemented soil primarily involves the nucleation effect and high reactivity of GO. These factors lead to a noticeable excitation effect on cement hydration, optimization of microstructure, and promotion of ion exchange. Simultaneously, the implementation of nanoscale GO optimizes gradation, filler effect, and bridging effect, thereby further promoting microstructure densification and resulting in enhanced water resistance and mechanical properties.

## Figures and Tables

**Table 1:** Chemical compositions and physical properties of plain loess.

Chemical composition	SiO_2_/%	Al_2_O_3_/%	Fe_2_O_3_/%	CaO/%	K_2_O/%
	65.17	16.22	3.56	4.01	2.24
Physical property	Liquid limit/%	Plastic limit/%	Index of plasticity	Optimum moisture content/%	pH
	30.2	18.5	11.7	12.30	7.2

**Table 2:** Chemical compositions of ordinary Portland cement P.O 42.5.

Composition	CaO/%	SiO_2_/%	Al_2_O_3_/%	MnO_2_%	Fe_2_O_3_/%	SO_3_/%	Loss on ignition/%
Proportion	61.60	22.60	4.98	2.32	2.90	2.31	1.81

**Table 3:** Chemical compositions of GO.

Composition	C/wt%	H/wt%	N/wt%	O/wt%	S/wt%	C/mol%	O/mol%	S/mol%
Proportion	45.27	2.70	0.07	47	2.09	55.664	43.372	0.964

**Table 4:** Proportion of cemented soil samples modified by GO.

Sample Name	Water Solid Ratio	Cement Content %	GO Content %
GCS-0.00	0.30	10	0.00
GCS-0.03	0.30	10	0.03
GCS-0.05	0.30	10	0.06
GCS-0.09	0.30	10	0.09
GCS-0.12	0.30	10	0.12

**Table 5:** GO particle size distribution.

Particle Size/μm	1	2	3	4	5	6	7	8	9
Volume ratio/%	4.47	9.50	12.85	17.04	19.55	15.36	9.78	8.10	3.35
Cumulative ratio/%	4.47	13.97	26.82	43.86	63.41	78.77	88.55	96.65	100.00

**Table 6:** Capillary absorption rate of modified soil–cement materials at different time intervals.

Sample	Water Absorption Rate of the Capillary (g/min)			
	0–60 min	60–180 min	180–360 min	360–480 min
GCS-0.00	0.902	0.238	0.021	0.005
GCS-0.03	0.753	0.207	0.021	0.004
GCS-0.06	0.710	0.184	0.017	0.005
GCS-0.09	0.596	0.157	0.012	0.003
GCS-0.12	0.701	0.181	0.013	0.004

## Data Availability

Data are contained within the article.
